# Impact of Co‐doping Eu^3+^ and Gd^3+^ in HMO‐Based Glasses for Structural and Optical Properties and Radiation Shielding Enhancement

**DOI:** 10.1002/bio.70045

**Published:** 2025-01-22

**Authors:** Ashwitha Nancy D'Souza, Sumanth G. Madivala, A. Vidya Saraswathi, M. I. Sayyed, M. Rashad, Sudha D. Kamath

**Affiliations:** ^1^ Department of Physics, Manipal Institute of Technology Manipal Academy of Higher Education Manipal India; ^2^ Department of Physics Acharya Institute of Graduate Studies (AIGS) Bangalore Soladevanahalli India; ^3^ Department of Physics Mahatma Gandhi Memorial College Udupi Kunjibettu India; ^4^ Department of Physics, Faculty of Science Isra University Amman Jordan; ^5^ Renewable Energy and Environmental Technology Center University of Tabuk Tabuk Saudi Arabia; ^6^ Advanced Materials Laboratory, Department of Physics, Faculty of Science University of Tabuk Tabuk Saudi Arabia

**Keywords:** build‐up factors, europium–gadolinium co‐doping, fast neutron removal, radiation shielding

## Abstract

Eu‐Gd co‐doped glasses composed of 15B_2_O_3_–12SiO_2_–(40‐x)TeO_2_–3Eu_2_O_3_–xGd_2_O_3_–12Bi_2_O_3_–8BaO–10ZnO with x = 0–4 mol% (coded as EuGd‐x) were fabricated using melt quench approach to develop transparent radiation shielding system. Their structural, optical and mechanical properties were examined. 5.3663, 5.4264, 5.7405, 5.4683 and 5.6756 g/cm^3^ were the densities of EuGd‐0, EuGd‐1, EuGd‐2, EuGd‐3 and EuGd‐4, respectively. Gamma shielding ability of EuGd system was simulated using the Photon Shielding and Dosimetry software in energy spectrum 0.015–15 MeV. On co‐doping Gd^3+^ with Eu^3+^, clear advancement in gamma and neutron shielding ability was perceived; EuGd‐2 dominates because of the highest density. At 0.662 MeV, the uppermost and lowermost (0.501 and 0.468 cm^−1^) linear attenuation coefficients (LAC) were demonstrated by EuGd‐2 and EuGd‐0, respectively. EuGd‐2 distinguished itself with least half value layer (HVL) = 1.383 cm at 0.662 MeV, compared to EuGd‐0 (1.483 cm). Photon build‐up inside the glasses declined with Gd concentration at low gamma energies (< 2 MeV). EuGd‐2 also showcased maximum fast neutron removal (FNR) cross‐sectional value (0.10935 cm^−1^). Comparison between singly doped Eu_2_O_3_, Gd_2_O_3_ glasses and the current co‐doped glasses established that EuGd‐2 is the superior gamma shield in terms of LAC, HVL and effective atomic number.

## Graphical abstract

EuGd‐2 glass has the highest FNR and lowest HVL values and hence is the best shielding agent for ionizing radiation.

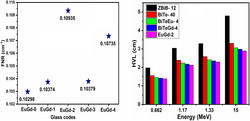

## 1 Introduction

Glasses are known for their exceptional properties like transparency, rust and moisture resistance, good fracture toughness and long durability. Adding heavy metal oxides (HMO) like PbO, Bi_2_O_3_, BaO, ZnO and TeO_2_ can alter the properties of glass by mainly increasing the density [1, 2]. Moreover, the glasses doped with rare earth oxides (RE) such as Eu_2_O_3_, Sm_2_O_3_ and Gd_2_O_3_ have shown promising results in the field of optoelectronics, non‐linear optics and solid‐state lighting devices due to high emission efficiency of 4f–4f electronic transitions [3, 4, 5]. RE oxides are widely known to enhance the density of glasses accompanied by improved thermal and structural properties. Growth of europium ions in the examined zinc tellurite glasses have led to the enhancement of their linear optical and gamma ray shielding factor [6]. Further, heavy element such as gadolinium is said to improve the density, transparency, refractive index and thermal and mechanical strength of the glasses. Gadolinium densifies the glass and plays a prominent role in detecting thermal neutrons and a good scintillator due to high solubility in the glass matrix.

The scientific and technical development in the regime of nuclear and radiation physics and its application in various sectors like power generation, medicine, diagnosis, treatment and also in the research laboratories lead to extreme discharge of ionizing radiation into the environment. Continuous exposure of these high‐energy radiation results in human organ damage, which can cause severe diseases like nausea, cancer and DNA structure alteration [7, 8]. Radiation awareness programmes have been implemented globally to tackle this situation, mainly on two classes. One is preparing and providing a suitable shield against radiation exposure. And the other is radiation dosimetry to effectively monitor the dose absorbed by the personnel and exposed to the environment. Conventionally, the walls made of lead and concrete along with composite materials like polymers and alloys are being used as radiation shielding materials [9, 10]. However, the walls or windows made up of glass material can dominate these materials overcoming their drawbacks such as transparency issues, complex preparation methods or lack of versatility in composition [11]. Recent attempts to replace the conventional shields with HMO‐based glasses have proven to be successful, and as an extension, the radiation blocking parameters are being evaluated for RE‐doped glasses too. There are few evidences in the literature where we find the enhanced ionizing shielding characteristics such as LAC, mass attenuation coefficient (MAC) and fast neutron removal (FNR) cross section with the successive addition of RE oxide concentration. One such observation was made by Zakaly et al. [3], where 4 mol% Sm_2_O_3_‐doped lithium borate glass produced the greatest MAC and LAC compared lower doping concentrations. Mostafa et al. [12] reported highest FNR values ranging from 0.0943 to 0.1130 cm^−1^, for lead–antimony borate glasses. Tellurite glasses containing RE oxides have been reported to attenuate gamma radiation comparably well with the ordinary concrete [13]. And tellurite glasses fundamentally allow highly concentrated doping of RE ions [14, 15, 16]. In our previous articles [17, 18], impressive results for gamma and neutron blocking efficiency have been reported for Eu^3+^‐doped and Gd^3+^‐doped B_2_O_3_–SiO_2_–TeO_2_–Bi_2_O_3_–BaO–ZnO glass system separately. For example, at 0.662 MeV, the compositions containing maximum mol% (4 mol%) of Eu_2_O_3_ and Gd_2_O_3_ have shown the greatest LAC values ranging from 0.46 to 0.47 and 0.46 to 0.48 cm^−1^, respectively. The combination of Eu and Gd in glasses has mostly been explored for spectroscopic investigations and luminescence properties for laser and LED applications [19, 20, 21, 22]. There is a lack of literature on Eu^3+^–Gd^3+^ co‐doped glasses analysed for radiation shielding application.

The current work is an attempt to investigate the improvement in tellurite glass properties when they are co‐activated with Eu^3+^ and Gd^3+^. A systematic analysis of physical, optical and mechanical and radiation shielding properties is presented. A simulation software called Photon Shielding and Dosimetry (Phy‐X/PSD) is utilized for obtaining theoretical values of important shielding parameters such as mass attenuation and linear attenuation coefficients‐ (MAC and LAC), half value layer (HVL), mean free path (MFP), effective and equivalent atomic number (Z_eff_ and Z_eq_) and gamma ray build‐up factors. The synthesized glasses were characterized for the theoretical FNR cross‐sectional values.

## 2 Materials and Methods

Europium–gadolinium co‐doped glasses with composition 15B_2_O_3_–12SiO_2_–(40‐x)TeO_2_–3Eu_2_O_3_–xGd_2_O_3_–12Bi_2_O_3_–8BaO‐10ZnO where x = 0, 1, 2, 3 and 4 mol% were synthesized using melt quench technique. The essential chemicals of high purity (> 99.9%) were precisely weighed and finely ground to get a homogeneous mixture, which is collected in alumina crucible. The mixture was melted inside a muffle furnace at the temperature of 1120°C for 3 h until a highly viscous liquid is formed. By quenching that liquid melt upon a slab maintained at 350°C temperature (below glass transition temperature), transparent glass samples were obtained. For getting rid of any thermal stress developed during melting, the glasses were annealed at constant temperature of 350°C for 2 h. The fabricated glasses were polished well to a thickness of 3 mm in Bainpol grinding/polishing machine using silicon carbide abrasive sheets. The glasses were coded as EuGd‐0, EuGd‐1, EuGd‐2, EuGd‐3 and EuGd‐4 in accordance with the Gd_2_O_3_ mol%. The densities of the synthesized glasses were determined using the density measurement kit of Contech analytical weighing balance (sensitivity = 0.001 g/cm^3^), which employs Archimedes' theory. Here distilled water was selected as immersion liquid. Density and the respective molecular weights were tabulated in Table 1. The x‐ray diffraction (XRD) characterization of the prepared glasses was done using Rigaku MiniFlex 600 XRD machine constituting of graphite monochromator and Cu–Kα radiation (40 kV and 15 mA) to be 5°–80° [23]. The FTIR data were recorded with FT/IR 6300 modelled JASCO FTIR spectrometer. Then the radiation shielding parameters were simulated using the Phy‐X/PSD software in the continuous gamma energy region of 0.015–15 MeV [24].

**TABLE 1:** Physical parameters of EuGd glasses.

Sample codes	EuGd‐0	EuGd‐1	EuGd‐2	EuGd‐3	EuGd‐4
Sample pictures	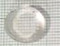	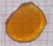	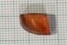	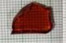	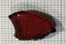
Average molecular weight, M (g/mol)	168.37	170.40	172.43	174.46	176.49
Density, ρ (g/cc) (±0.01)	5.3663	5.4264	5.7405	5.4683	5.6756
Molar volume, V_M_ (cm^3^)	31.3753	31.4017	30.0370	31.9032	31.0955
Number density of Gd^3+^ ions in host glass, N_Gd_ (×10^23^ ions/mol)	0	1.918	4.009	5.662	7.464
Polaron radius, P_r_ (nm)	0	99.883	54.4651	48.711	43.879
Inter‐ionic distance between Gd^3+^ ions, I_r_ (nm)	17.341	13.561	12.087	17.341	10.888

## 3 Results and Discussion

### 3.1 Physical and Structural Studies

From the analysis of various physical parameters, the effect of co‐doping Gd^3+^ ions with Eu^3+^ on the network structure of the glasses can be characterized. The experimentally measured densities (ρ) were listed in Table 1. The parameters like molar volume V_M_, number density N_Gd_ of Gd^3+^, polaron radius P_r_ and interionic distance I_r_ were tabulated in Table 1. The following equations were utilized to determine the above‐mentioned parameters:

(1) $$\mathrm{VM}=\sum_{i}x_{i}\;\frac{M_{i}}{\rho }$$

(2) $$\mathrm{NGd}=\left(\%\mathrm{mol}\;\text{of}\;\mathrm{RE}\right)\left(\frac{N_{A\;}\rho }{M}\right)$$

(3) $$\mathrm{Pr}=\left(\frac{1}{2}\right)\sqrt[{3}]{\frac{\pi }{6N_{\mathrm{Gd}}}}$$

(4) $$\mathrm{Ir}=\sqrt[{3}]{\frac{1}{N_{\mathrm{Gd}}}}$$

Here, x_i_ and M_i_ are the mole fractions and molecular weights of the *i*th component, respectively; ρ is density of the glass, N_A_ being Avogadro's number; and M = $\sum M_{i}x_{i}$ is the molecular weight.

It was observed that there was enhancement in the density of samples with the increase in Gd_2_O_3_ concentration till EuGd‐2. At 3 mol% Gd_2_O_3_ concentration, we identified a sudden decrease in density, which may have occurred due to Gd–O–Te or Gd–O–B bond creation, which might have weakened cross‐linking inside the glass network. The molar volume does not follow any definite pattern with the Gd^3+^ doping. It was found that EuGd‐2 has the lowest molar volume (30.307 cm^3^/mol). The parameters such as number density (N) and field strength (F) increased with the rise in molecular weight, which pointed towards the positive influence of Gd_2_O_3_. But the other parameters such as polaron radius and inter‐ionic distance exposed a diminishing pattern that can be associated to the soaring compactness of glass through successive inclusion of Gd^3+^ ions [25]. Oxygen packing density (OPD) followed the same path as density.

**FIGURE 1:**
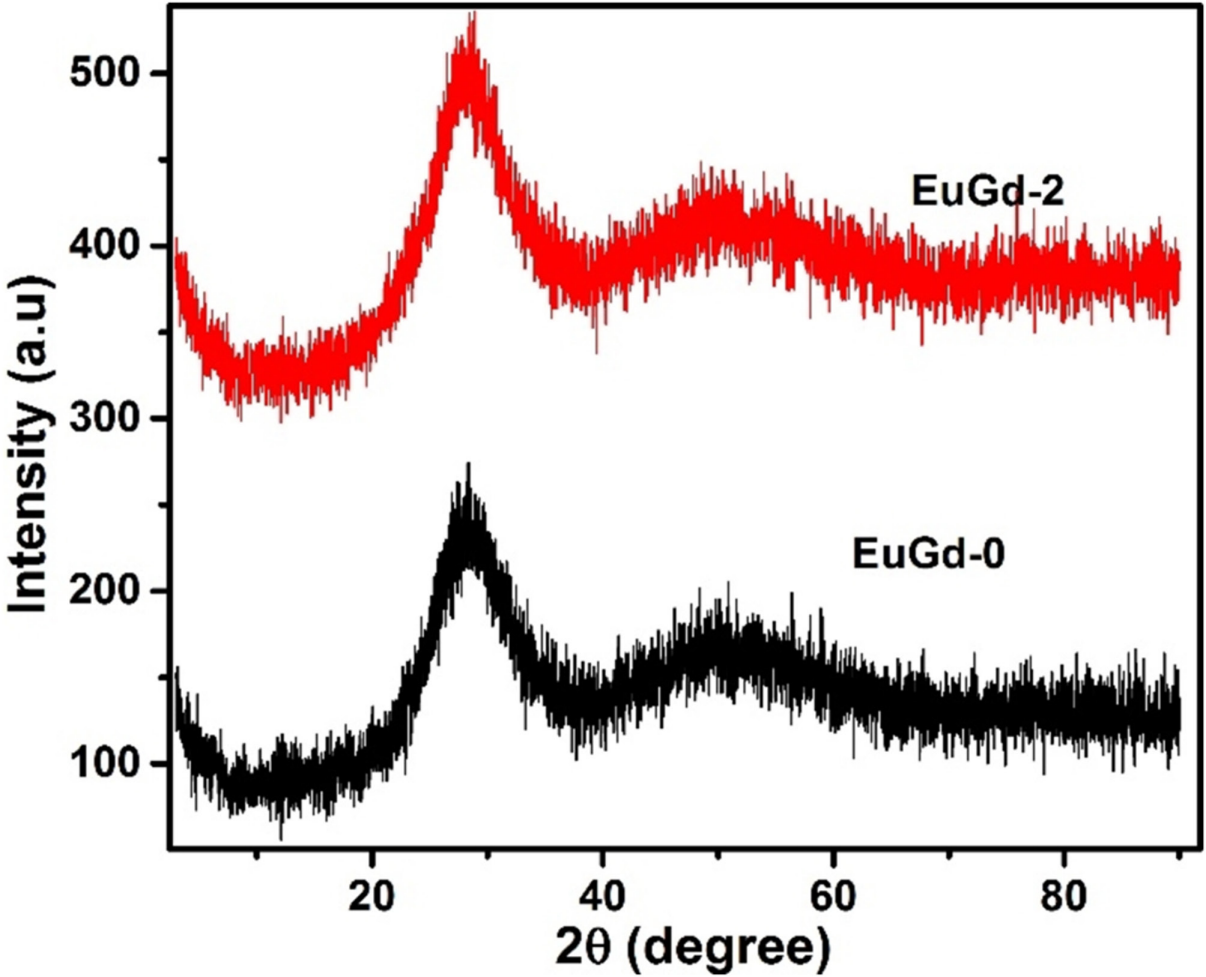
XRD patterns of EuGd glasses.

The recorded XRD patterns for the sample glasses (EuGd‐0 and EuGd‐2) are displayed in Figure 1. The absence of any characteristic crystalline peaks in the patterns confirmed that co‐doped glasses continued to exist as amorphous material [26].

**FIGURE 2:**
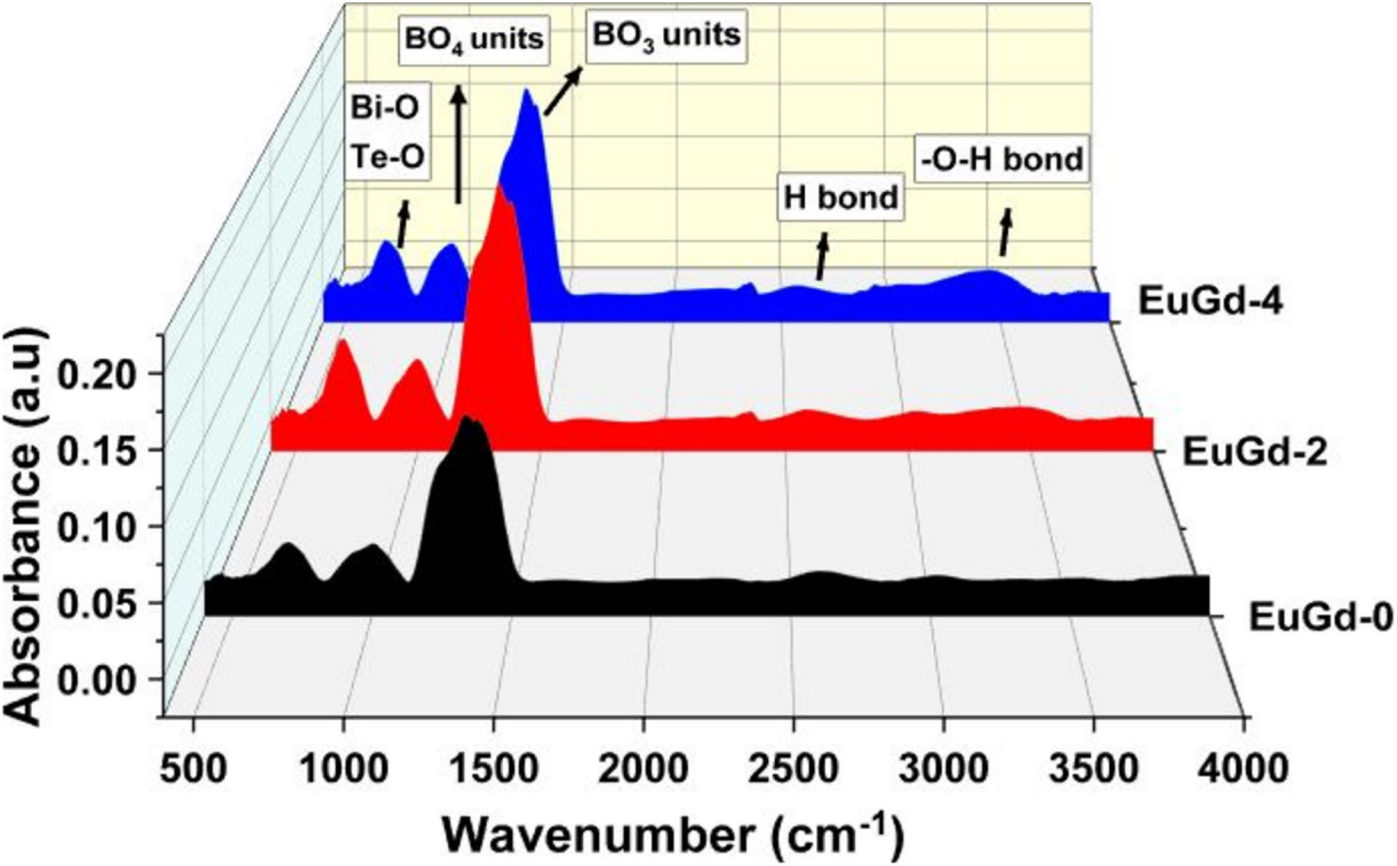
FTIR spectra recorded for the Eu^3+^–Gd^3+^ co‐doped glasses in 400–4000 cm^−1^ wavenumber region.

The effect of co‐doping on the essential structural units of the host boro‐tellurite glass was investigated by recording the FTIR spectra for EuGd‐0, EuGd‐2 and EuGd‐4 glasses in the 400–4000 cm^−1^ wavenumber range (Figure 2). All the three glasses showed the existence of absorption bands corresponding to HM–O, B–O, Si–O and Te–O linkages. The bands in 400–600 cm^−1^ spectrum region corresponds to vibrational bands of metal oxides/HMO. The frail absorption bands at 410–470 cm^−1^ range were associated with bending vibrational bands of Te–O–Te and Bi–O–Bi and Bi–O linkages in TeO_4_ and BiO_6_ structural units. Additionally, the bands linked to symmetric stretching vibrations of Te–O or Bi–O bonds in the pyramidal TeO_3_ or BiO_3_ units were found in 600–800 cm^−1^ range. But the bands linked to stretch vibrational bands of B–O and Si–O linkages in tetrahedral BO_4_ and SiO_4_ units were present in 800–1200 cm^−1^ region. The bands formed in 1200–1550 cm^−1^ region corresponds to asymmetric stretching vibrations of B–O linkages in meta‐, pyro‐ and orthoborate groups of BO_3_ units. The absorption band belong to hydroxyl group vibrations found at non‐bonding oxygen sites occurring around 2300 and at 2600 cm^−1^ were associated with hydrogen bonding. The presence of water molecule in the glass structure is confirmed by the peaks in 3200–3600 cm^−1^ region [27].

### 3.2 Radiation Shielding Property of Eu^3+^–Gd^3+^‐Doped Glasses

**FIGURE 3:**
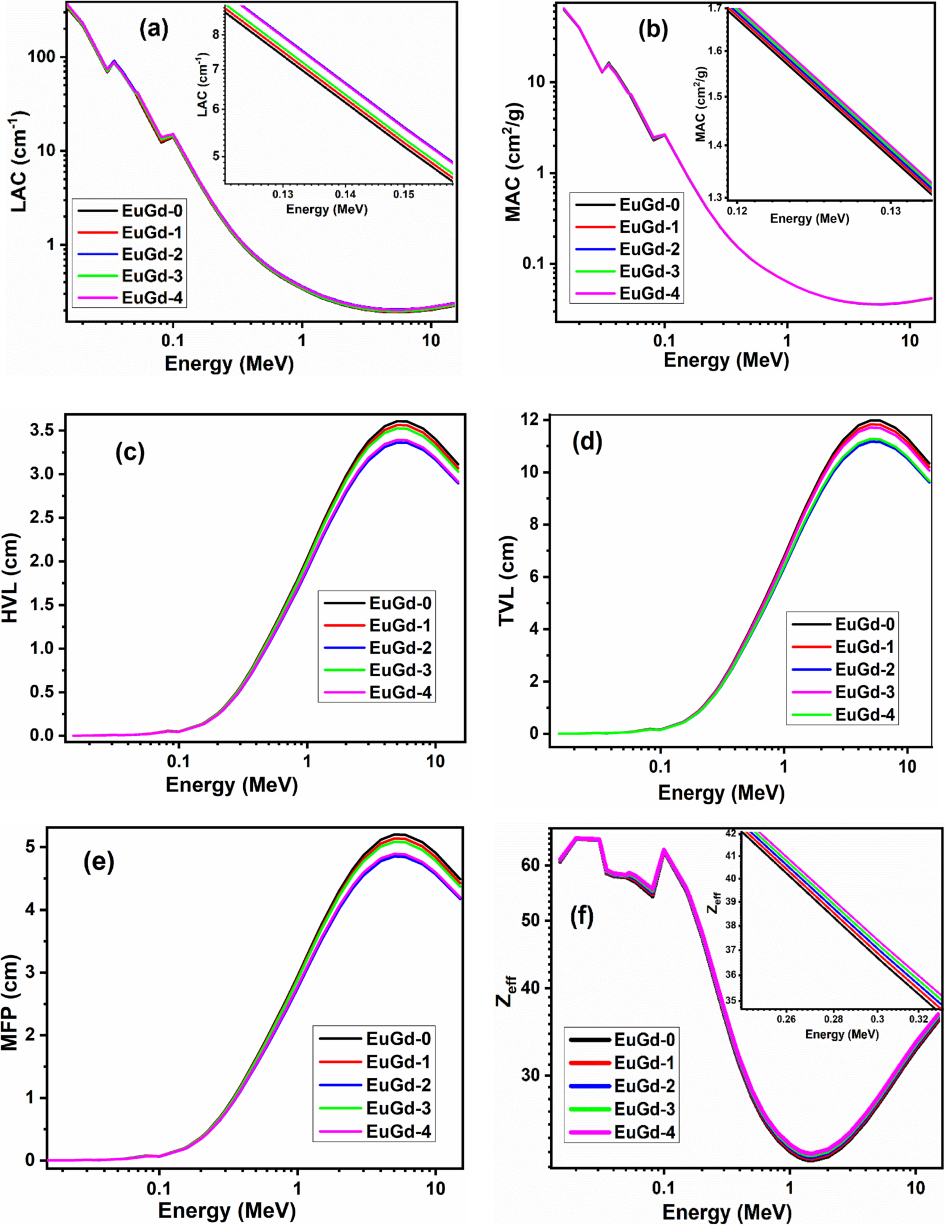
The graphs of radiation shielding parameters against energy in 0.015–15 MeV for EuGd glass samples.

The theoretical simulation of radiation shielding parameters for the synthesized samples was done with Phy‐X/PSD for the wide energy range of 0.015–15 MeV [24]. The graph of these parameters versus energy of gamma rays was plotted for the current Eu–Gd co‐doped glasses and shown in Figure 3. The theoretical relations of these shielding parameters are given using Lambert–Beer law, which is formulated as

(5) $$I=I_{0}e^{-\mathrm{\mu t}}$$

Here, I and I_0_ are the radiation intensities with and without the shield material, respectively. t is the thickness of the sample and μ denotes LAC. MAC is the parameter that signifies the interaction (absorption/scattering) cross section of gamma photons with the shielding medium.

(6) $$\mu_{m}=\frac{\mu }{\rho }$$

HVL is an important shielding quantity that gives the thickness of the shield required to get half of the initial intensity of the incident radiation. HVL is derived from Lambert–Beer's law as follows:

(7) $$\mathrm{HVL}=\frac{\ln2}{\mu }=\frac{0.693}{\mu }$$

Similarly, tenth value layer (TVL) provides the thickness required to lower the incident photon intensity to 1/10th of its value. The TVL is computed by the equation below.

(8) $$\mathrm{TVL}=\frac{\ln10}{\mu }=\frac{2.303}{\mu }$$

MFP measures the average distance between the successive photon–particle interactions and is given by

(9) $$\mathrm{MFP}=\frac{1}{\mu }$$

Effective atomic number gives the collective atomic number of a composite material and is calculated using the formula

(10) $$\text{Zeff}=\frac{\sigma_{a}}{\sigma_{e}}$$

Here, $\sigma_{a}$ and $\sigma_{e}$ are total atomic and electronic cross sections [24].

The higher value of LAC for a shielding material signifies strong photon interaction with the material [28]. In the graph of Figure 3a, the Eu^3+^–Gd^3+^ co‐doped glasses were taken for LAC analysis in 0.015–15 MeV region, and it was observed that the LAC value was greater for lower photon energy of 0.015 MeV. We also observed two sudden peaks at 0.04 and 0.1 MeV, which are the K‐edges for Bi^3+^ and Ba^2+^ ions, respectively [29]. With the energy, the LAC value decreased exponentially for all the samples. Among the present samples, EuGd‐2 showed the highest LAC value in the entire energy spectrum compared to other samples owing to its greatest density value. However, the LAC of EuGd‐4 > EuGd‐3 > EuGd‐1 > EuGd‐0. Thus, co‐doping with Gd^3+^ surely improved the radiation attenuation ability of the Eu^3+^‐doped glasses; EuGd‐2 offered maximum shielding effect. The graph of MAC against energy was plotted for the Eu–Gd co‐doped glasses as displayed in Figure 3b. The MAC values followed the same trend as LAC with energy as well as composition. Besides, we observed the similar two peaks as in Figure 3a, which are also the K‐edges of Bi and Ba ions [30].

In general, the smaller the HVL of a shielding agent, the greater is the radiation blocking capacity of that material as far as thickness requirement is concerned. In Figure 3c, it was observed that the HVL value increases exponentially with respect photon energy in 0.1–6 MeV region. And among the synthesized samples, EuGd‐2 has the minimum HVL value at each energy. We point out that the HVL of EuGd‐0, EuGd‐1, EuGd‐2, EuGd‐3 and EuGd‐4 glasses at 0.662 MeV are 1.48, 1.46, 1.38, 1.45 and 1.39 cm respectively. Conclusively, EuGd‐2 can provide better shielding effect with the least thickness in comparison to other samples. Similarly, the graph of TVL versus photon energy is presented in Figure 3d. The TVL values of EuGd‐0, EuGd‐1, EuGd‐2, EuGd‐3 and EuGd‐4 glass samples at 0.662 MeV were 4.92, 4.86, 4.59, 4.82 and 4.64 cm, respectively. Therefore, EuGd‐2 glass of mere thickness 4.59 cm is sufficient to attenuate the standard 0.662 MeV radiation photon to 1/10th of its initial intensity. From this, we again come to the deduction that EuGd‐2 is the best co‐doped composition for radiation shielding.

MFP is another shielding parameter whose value should be small enough to get a sample having maximum photon interaction ability. From Figure 3e, we observed that MFP value increased with photon energy (0.018–6 MeV) and diminished with Gd^3+^ doping up to 2 mol%. Among the synthesized glass samples, the EuGd‐2 provided minimum MFP value. Thus, the MFP results also confirmed the less thickness requirement of EuGd‐2 to provide better radiation shielding. The graph of Z_eff_ against photon energy has been presented in Figure 3f. Here, Z_eff_ showed random pattern with initial increase in photon energy. From 0.1 to 1.5 MeV, Z_eff_ decreased exponentially. At the end of the spectrum, there was a slight rise in Z_eff_ values up to 15 MeV. Among the five tested samples, the EuGd‐4 glass produced highest Z_eff_ value. This may be due to the increase in the molar concentration of Gd_2_O_3_ and increased average molecular weight [31].

**FIGURE 4:**
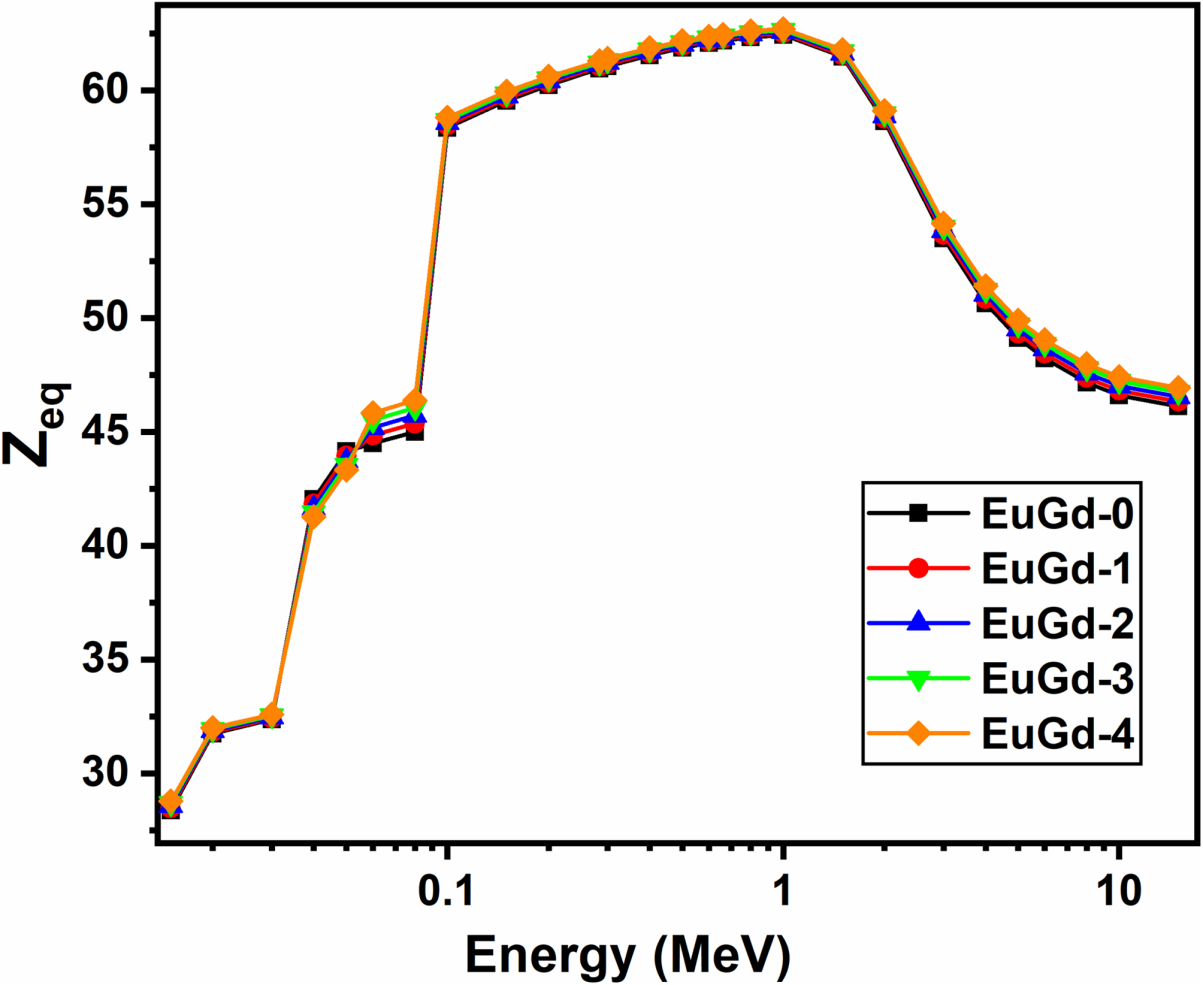
Equivalent atomic number of EuGd glasses in 0.015–15 MeV range.

Z_eq_ or equivalent atomic number is one such glass shielding parameter that explains the property of glass with equivalent elements same as single element's atomic number. Z_eq_ is the tool to understand the ϒ‐ray build‐up factor of the glass through Compton scattering process. The PSD software adopts interpolation technique to calculate the Z_eq_ for the synthesized glasses in 0.015–15 MeV range as per the theoretical equations provided in literature [24, 32]. The plotted graph of Z_eq_ versus photon energy is shown in Figure 4 where we find that with the increase in Gd_2_O_3_ concentration, the Z_eq_ increased continuously at every energy with EuGd‐4 glass exhibiting the maximum value. The maximum Z_eq_ for EuGd‐4 is due to higher weight per cent of Gd in it, resulting in greater molecular weight. The Z_eq_ was found to be high at both lower and higher end of energy spectrum (< 0.1 MeV and > 3 MeV) for all the samples. This is an indicator that photoelectric effect and pair (e^−^ and e^+^) production processes are playing major role in these energy regions. In the intermediate or Compton scattering region, the Z_eq_ is found to be maximum.

**FIGURE 5:**
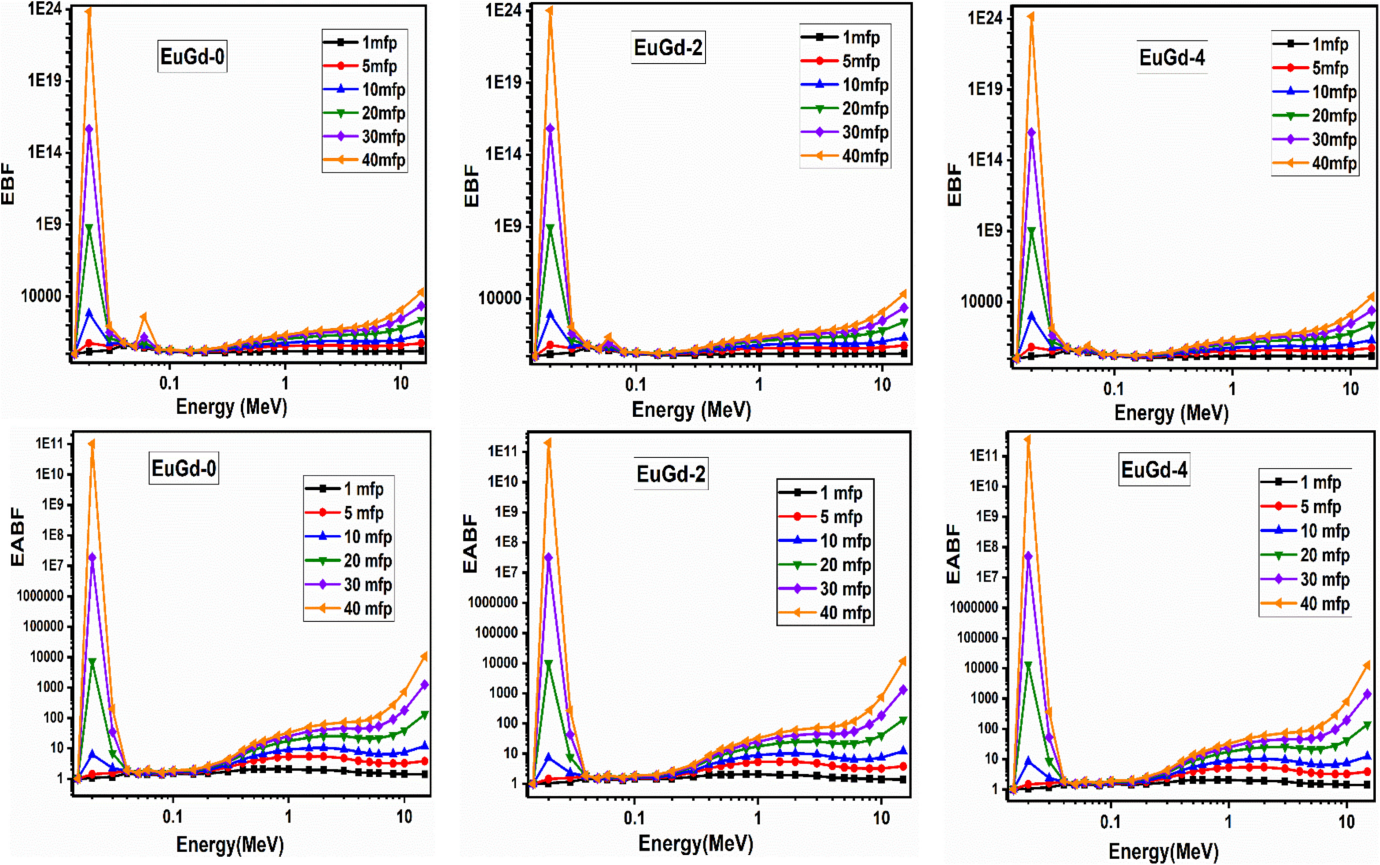
Graph of EBF and EABF of the selected EuGd glasses in 0.015–15 MeV region.

Following the Z_eq_ computation, the PSD software interpolated the build‐up factor using G‐P or geometric progression fit parameters in 0.015–15 MeV region. The details of the interpolation formula are explained reference articles [33, 34]. Figure 5 graphically represents the exposure and energy absorption build‐up factor (EBF and EABF) values of the selected Eu–Gd co‐doped glasses at different penetration depths (1–40 mfp range). In the graphs, we see that gamma build‐up increased continuously with the penetration depth in case of all the samples. This is an implication of the enhanced photon build‐up inside the shielding material due to multiple photon scattering processes. The peaks located at 0.02 and 0.06 MeV were associated with L_I_‐absorption edge of bismuth and K‐edges of tellurium [35]. The EBF values were found to be greater than EABF for the corresponding glass composition, which suggested that more photons were scattered rather than absorbed, therefore losing most of their energy and remaining inside the material for longer time. Moreover, the three‐photon interaction processes influence the photon build‐up. In Figure 5, we observed lesser EBF and EABF values at low energies due to photoelectric effect. At the medium photon energies, the build‐up factors attained higher values, but the photons were not entirely absorbed. But large EBF and EABF were found in the high photon energy range owing to the complete photon absorption by pair production process [36].

**FIGURE 6:**
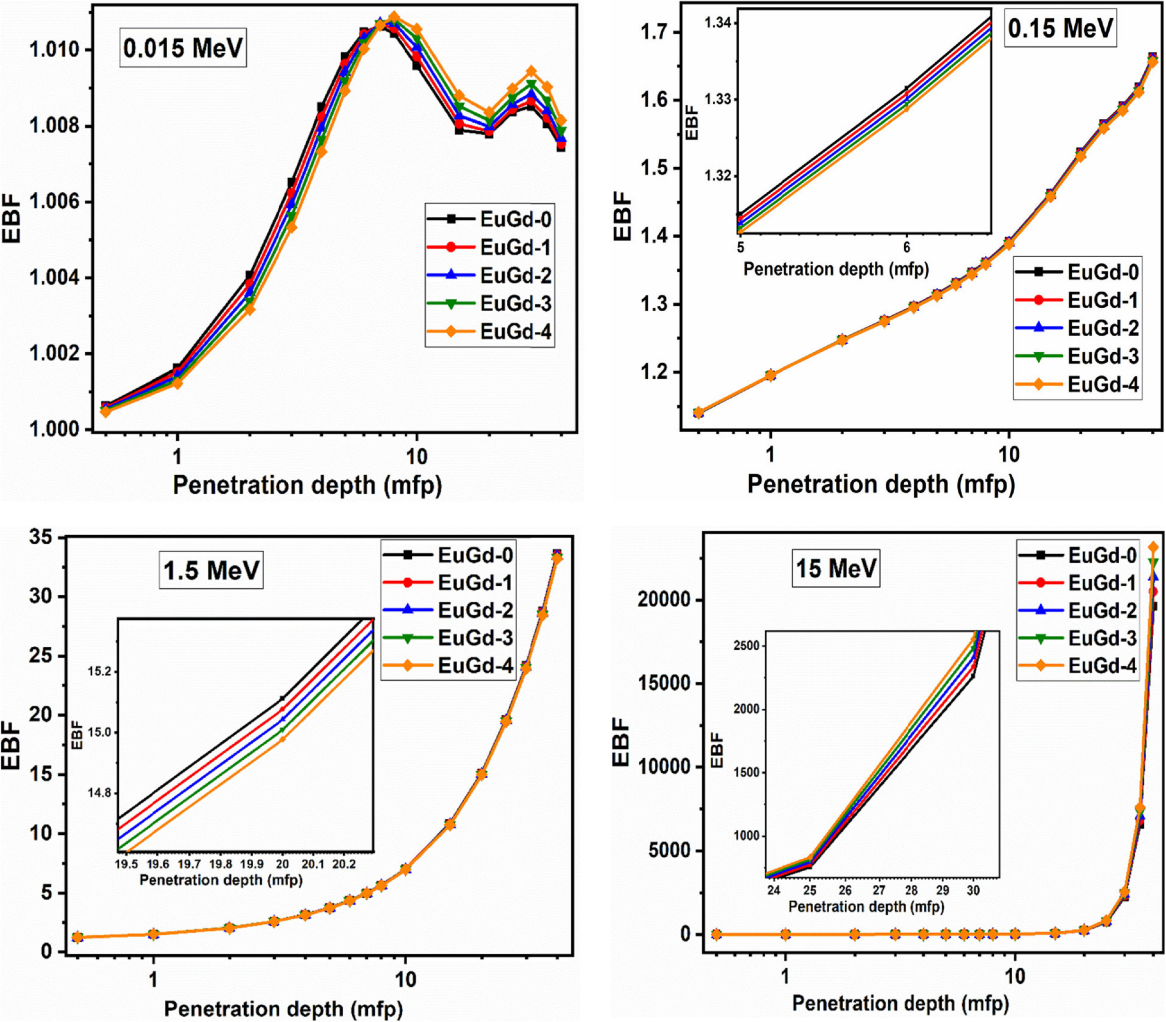
EBF values plotted against penetration depth for EuGd glasses at selected energies.

The graphs in Figure 6 provide the graphs of EBF of the prepared glass materials at 0.015, 0.15, 1.5 and 15 MeV energies in 0.5–40 mfp range of penetration depth. It was observed that EBF reduced with Gd_2_O_3_ concentration at low (0.015 and 0.15 MeV) and medium energies (1.5 MeV). But EBF amplified with Gd content for photon energy > 2 MeV. In the 0.015 MeV graph, penetration depths < 8 mfp corresponded to descending BF with Gd^3+^ concentration, whereas depths > 8 mfp led to reversed result, with EuGd‐4 developing maximum build‐up. At 0.15 and 1.5 MeV, the EuGd‐4 displayed minimum gamma build‐up in the entire penetration depth range. However, the pair production range of energy 15 MeV weakened the BF effect with Gd^3+^ content. Similar effect of Gd^3+^ doping on the glass system was observed in other research works [37, 38]. One of the essential qualities of a good shielding material is low photon build‐up. Therefore, co‐doping Gd^3+^ to the Eu_2_O_3_ glass is effective in achieving good ϒ‐ray blocking property.

The fast neutron shielding ability of the current co‐doped glasses is also evaluated in this study by determining the theoretical value of a parameter known as FNR cross section or Σ_r_ (expressed in cm^−1^). The mixture rule used for the calculation is as follows:

(11) $$\sum_{r}=\sum_{i=1}^{n}{\left(\rho_{x}\right)}_{i}{\left(\sum_{r/\rho }\right)}_{i}$$

**FIGURE 7:**
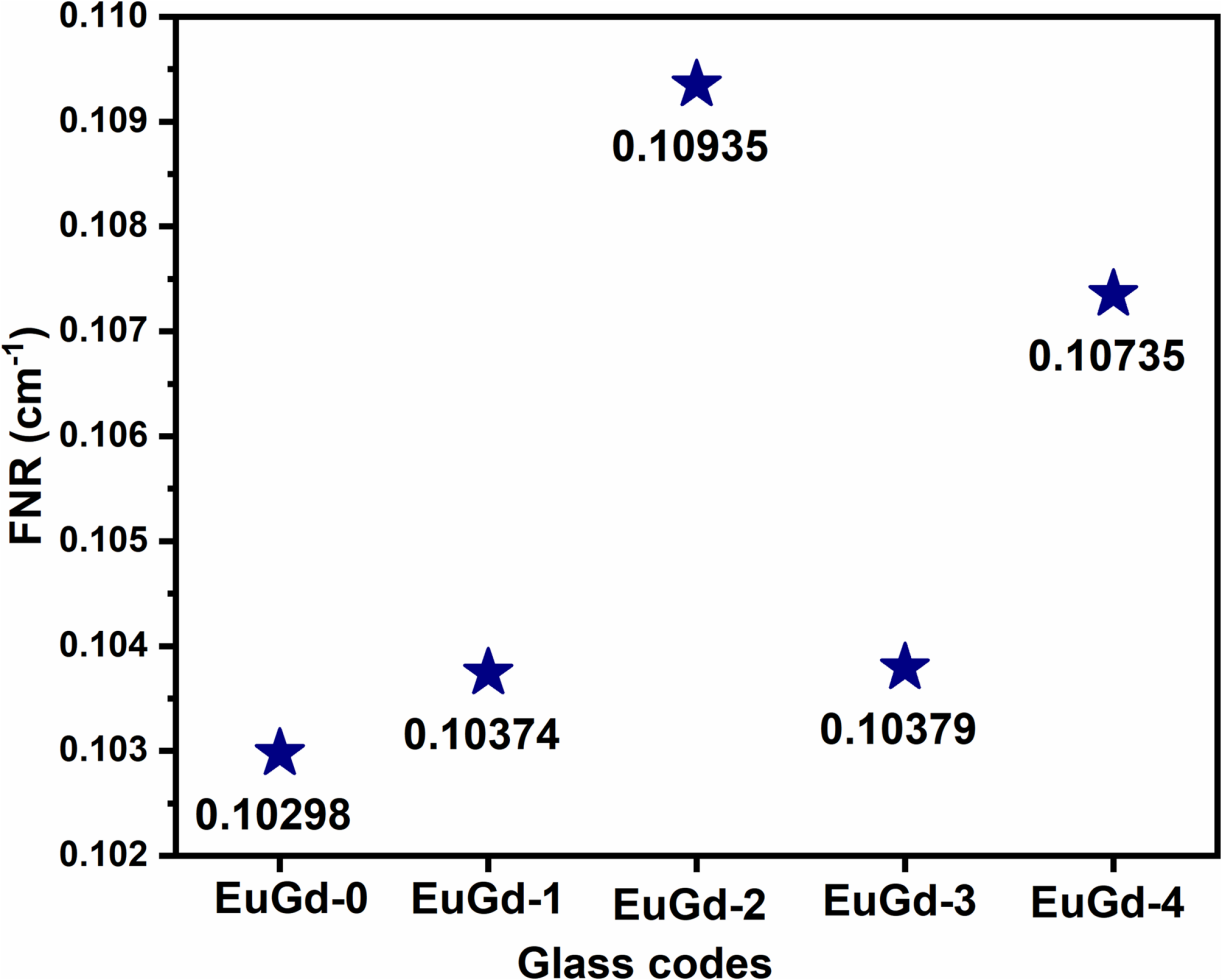
FNR values of EuGd glasses*.*

Here, $\rho_{x}$ denotes partial density, and the parameter Σ_r/ρ_ is known as mass removal cross section (expressed in cm^2^/g). The Σ_r/ρ_ value corresponds to constituent elements, and the Σ_r/ρ_ values for all elements are compiled in NCRP‐1957 report and other sources [39, 40, 41, 42]. The computed FNR for the Eu^3+^–Gd^3+^ co‐doped glasses are shown in Figure 7. Here, EuGd‐2 glass again showed dominance in FNR probability with respect to other prepared glasses by producing maximum FNR = 0.10935 cm^−1^. Compared to the singly doped Eu^3+^ host glass, the co‐doped glasses revealed definite improvement in fast neutron shielding as evident from Figure 7. This could be attributed to the high thermal and fast neutron cross‐sectional property of gadolinium [43, 44]. Also, the FNR value of the current glasses are certainly higher than that of conventional shielding agent, that is, ordinary concrete (0.093 cm^−1^) [45].

### 3.3 Radiation Shielding Comparison of HMO‐ and RE‐Doped Glasses

In our previous works [18, 46, 47, 48], four glass systems with the following compositions were investigated with respect to their radiation shielding proficiency.

- ZBiB glasses: (60‐x_1_) B_2_O_3_–20SiO_2_–x_1_ Bi_2_O_3_–12ZnO–8BaO with x_1_ = 0, 2, 4, 6, 8, 10 and 12 mol%.
- BiTe glasses: (50‐x_2_) B_2_O_3_–17.5SiO_2_–x_2_ TeO_2_–12ZnO–12Bi_2_O_3_–8BaO–0.5CeO_2_ with x_2_ = 0, 10, 20, 30 and 40 mol%.
- BiTeEu glasses: 12B_2_O_3_–16 SiO_2_–x_3_ Eu_2_O_3_–(40‐x_3_)TeO_2_–12Bi_2_O_3_–12ZnO–8BaO with x_3_ = 0, 1, 2, 3 and 4 mol%.
- BiTeGd glasses: 12B_2_O_3_–16 SiO_2_–x_4_ Gd_2_O_3_–(40‐x_4_) TeO_2_–12Bi_2_O_3_–12ZnO–8BaO with x_4_ = 0, 1, 2, 3 and 4 mol%.

**FIGURE 8:**
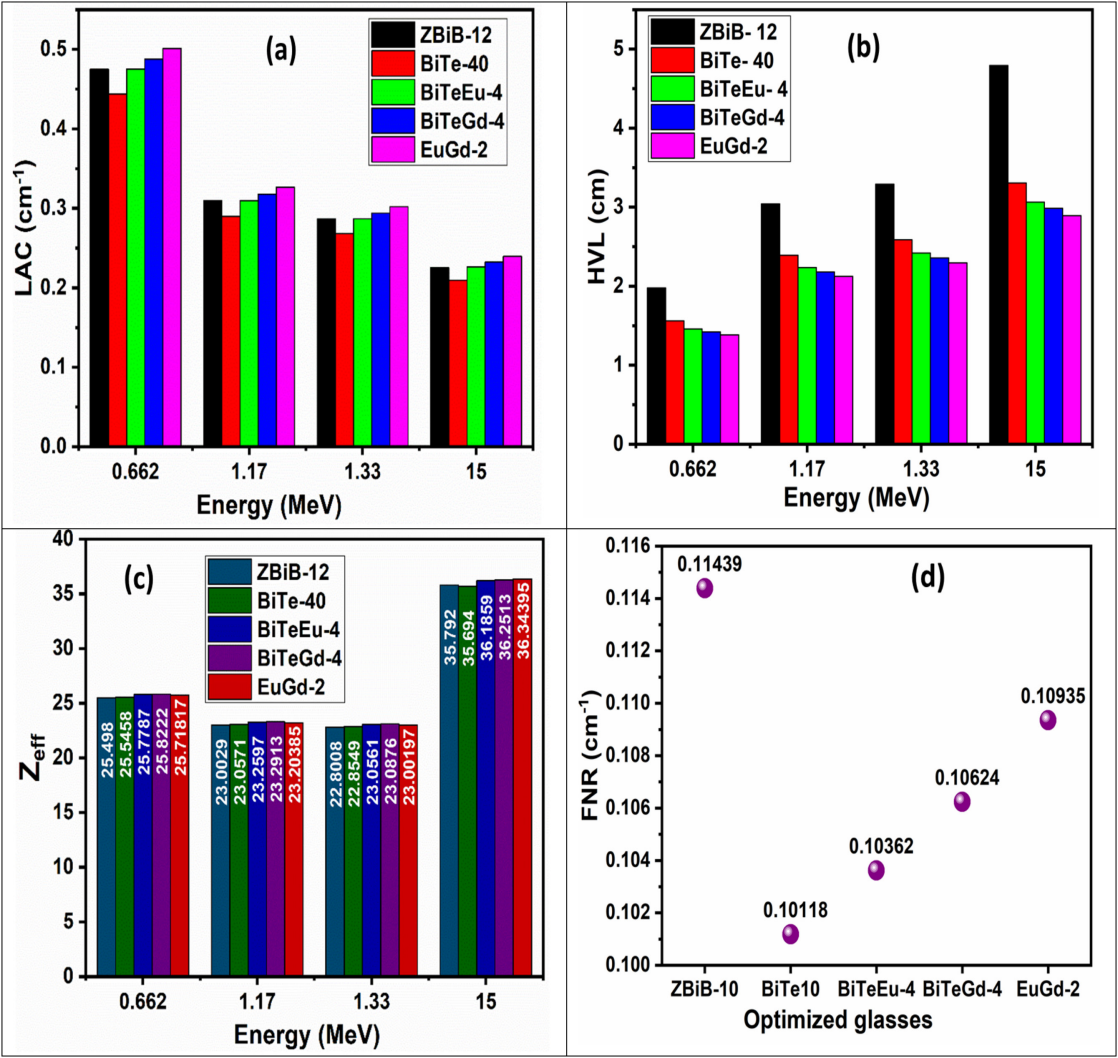
Graphs representing the (a) LAC, (b) Z_eff_, (c) HVL and (d) FNR of the HMO and RE added glasses.

By evaluating the gamma shielding parameters such as MAC, LAC, Z_eff_, HVL and MFP both theoretically and experimentally, the optimum glass samples in the above four systems for radiation blocking application were concluded to be ZBiB‐12, BiTe‐40, BiTeEu‐4 and BiTeGd‐4, respectively. The basis of this optimization was the highest value of MAC, LAC and Z_eff_ accompanied with the minimum values of HVL and MFP among the glasses of same system [18, 46, 47, 48]. The compositions of the four glass systems were decided such that the optimum glass from ZBiB system (ZBiB‐12) is taken and added with another HMO TeO_2_, resulting in BiTe glass system. BiTe‐40, which is the optimized glass sample of BiTe series, is added with different concentrations of Eu_2_O_3_ and Gd_2_O_3_ to obtain BiTeEu and BiTeGd glass systems. In the present work, the co‐doping of Eu_2_O_3_ and Gd_2_O_3_ was attempted by taking fixed amount of 3 mol% of Eu_2_O_3._ The column graphs in Figure 8a–c give the comparison of LAC, HVL and Z_eff_ parameters of ZBiB‐12, BiTe‐40, BiTeEu‐4 and BiTeGd‐4 glasses with the present EuGd‐2 glass. However, the optimum glass for fast neutron shielding in the case of ZBiB system was ZBiB‐10, and the comparison of FNR values of all the optimum glasses with EuGd‐2 glass is shown in Figure 8d.

From the LAC plots in Figure 8a, we see that singly doped BiTeGd‐4 glass and co‐doped EuGd‐2 glass showed higher LAC values at the selected energies; EuGd‐2 exhibiting the greatest attenuation compared to other compositions revealed that it is the best possible glass for gamma blocking. The HVL graphs in Figure 8b exhibited orderly decrement in HVL as EuGd‐2 < BiTeGd‐4 < BiTeEu‐4 < BiTe‐40 < ZBiB‐12. Therefore, EuGd‐2 glass has the ability to block the gamma radiation with the least thickness at all the energies. The Z_eff_ of all the optimum samples were almost same with comparatively highest range exhibited by BiTeGd‐4 glass as shown in Figure 8c. Figure 8d depicted that ZBiB‐10 is still the sample with highest FNR capacity. However, the FNR of BiTe‐40, BiTeEu‐4, BiTeGd‐4 and EuGd‐2 followed an almost linear path with co‐doped glass producing maximum FNR. If we compare the values with conventional shields such as ordinary concrete (Σ_r_ = 0.093 cm^−1^) [45] and binary glass systems such as lead borate (Σ_r_ = 0.11 cm^−1^) [45] and bismuth borate (Σ_r_ = 0.1134 cm^−1^) [49], then BiTe‐40, BiTeEu‐4, BiTeGd‐4 and EuGd‐2 glass are able to surpass the FNR of ordinary concrete, whereas ZBiB‐10 is superior to all the above.

The overall shielding proficiency of the glasses can be judged by comparing the HVL values shown in Figure 8c with the two important concrete shields, namely, barite and ferrite concretes, which are used conventionally. At 1.332 MeV, the HVL of barite concrete is 3.75 cm, and ferrite concrete is 2.75 cm [50], both values being higher than HVL of BiTe‐40, BiTeEu‐4, BiTeGd‐4 and EuGd‐2 glasses. So, we can say that all the four optimized glasses are better gamma shields than conventional concrete materials. Another comparison says that a set of binary bismuth borate glasses reported by Kaewkhao et al. [51] produce HVL values in 1.9–1.4 cm range and Z_eff_ values in 10–20 range. The authors also mentioned that binary lead borate glass system exhibited HVL values in 2.25–1.75 cm range at 0.662 MeV. Therefore, it can be summarized that the synthesized BiTe‐40, BiTeEu‐4, BiTeGd‐4 and EuGd‐2 glasses showed better gamma shielding property than binary bismuth and lead borate glasses [51].

### 3.4 Optical Studies

Trivalent rare earth (RE^3+^) doped oxide glasses are well known for their impressive optical applications. Because of this, optical parameters such as refractive index n, dielectric constant ε, molar refractivity R_m_, molar electronic polarizability $\alpha_{m}$ and optical basicity Λ have been studied in this research work using the equation below:

(12) $$n=\sqrt{0.545\rho +1}$$

(13) $$\varepsilon =n^{2}$$

(14) $$\mathrm{Rm}=\left(\frac{n^{2}-1}{n^{2}+2}\right)V_{m}$$

(15) $$\alpha_{m}=\frac{3}{4\pi N_{A}}R_{m}$$

(16) $$\Lambda =\sum_{i}x_{i}\Lambda_{i}$$

Here, V_M_ is the molar volume, and the value $\frac{4\pi }{3}$ is represents the constant in Lorentz function [52]; $\Lambda_{i}$ is optical basicity of constituent oxide molecule [53]. The resulting optical parameters values are tabulated in Table 2. Addition of Gd^3+^ ions to the Eu^3+^‐doped glass network resulted in the development of densely packed structure of RE modifiers in the host material, consequently increasing refractive index. At 3 mol% addition of Gd_2_O_3_, the molar concentration of Eu_2_O_3_ nearly equals that of Gd_2_O_3_. This may be the reason for reduction of n value [54]. Due to the direct relation between refractive index, polarizability and optical basicity, the latter quantities observe an increasing trend. The increment in optical basicity refers to the increase in the capacity of transferring electron by the oxide ions to the neighbouring cation [55].

**TABLE 2:** Optical parameters of EuGd glasses.

Sample codes	RIN	Dielectric constant ɛ	*R*_m_ (cm^3^/mol)	*R* (%)	Mol. electron polarizability ε_m_ (Å^3^)	Optical basicity ᴧ
EuGd‐0	1.9811	3.9246	15.4881	0.9625	6.1461	0.912
EuGd‐1	1.9893	3.9574	15.5885	0.9788	6.1859	0.9155
EuGd‐2	2.0319	4.1286	15.3336	1.0648	6.0848	0.896
EuGd‐3	1.9951	3.9802	15.8988	0.9901	6.309	0.929
EuGd‐4	2.0232	4.0932	15.7856	1.0469	6.2641	0.9191

**FIGURE 9:**
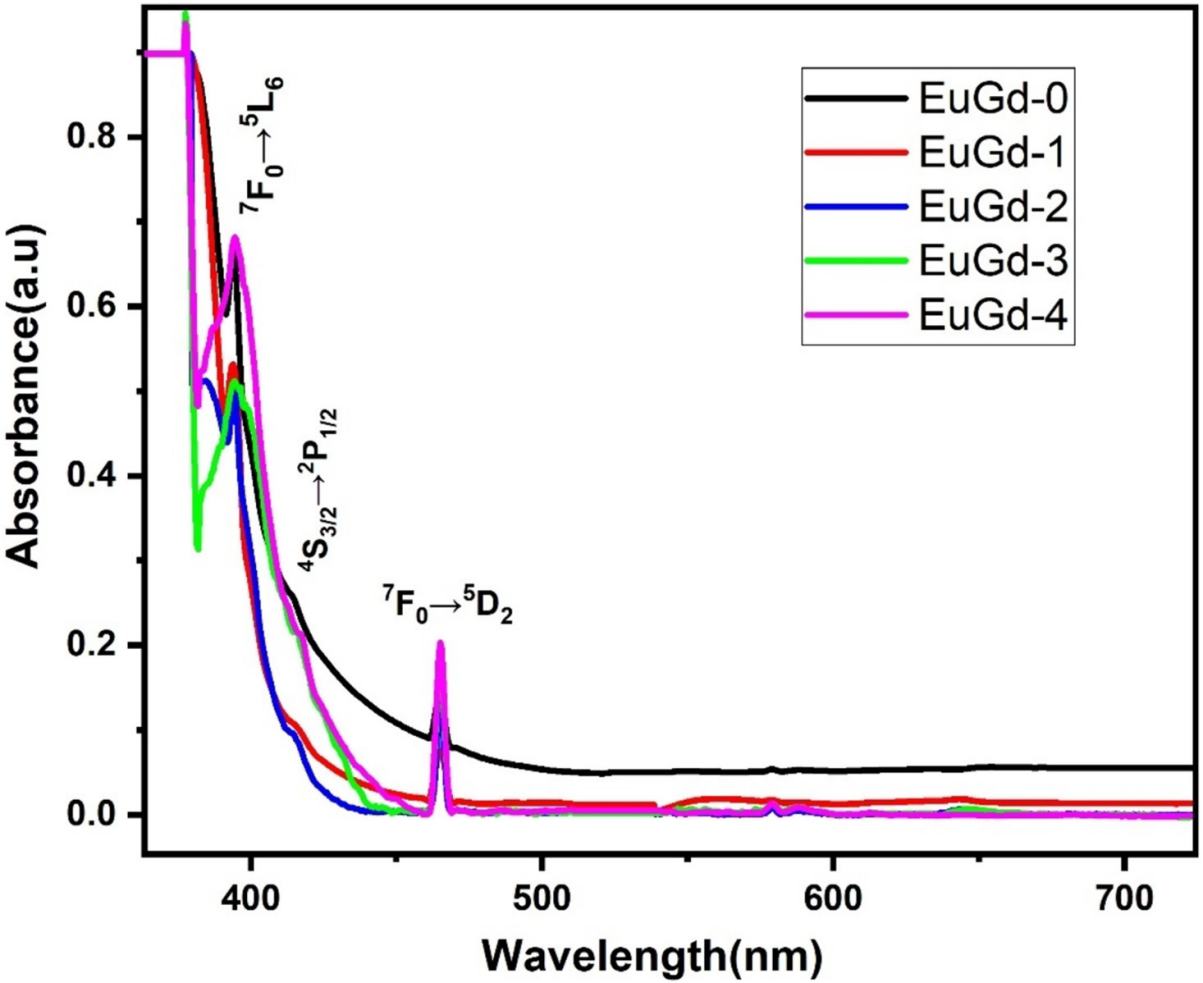
UV–visible absorption spectra of EuGd glasses in 350–750 nm wavelength region.

The absorption spectra of EuGd samples were documented in the UV–visible region (350–750 nm) and presented in Figure 9. The spectra showed the presence of three bands at around at 394, 416 and 465 nm. The peaks at 394 and 465 nm follows the respective transitions ^7^F_0_ → ^5^L_6_ and ^7^F_0_ → ^5^D_2_ of Eu^3+^ ions [56]. But the peak at 416 nm corresponds to ^4^S_3/2_ → ^2^P_1/2_ transition of Bi^3+^ ions [57]. The transition ^7^F_0_ → ^5^L_6_ (394 nm) shows high intensity because such transition is forbidden by the selection rule for ΔS → ΔL but allowed by the selection rule for ΔJ. The induced electric dipole transition ^7^F_0_ → ^5^D_2_, which is present at 465 nm, is hypersensitive in nature [58].

### 3.5 Mechanical Parameters

The shielding glasses should be able to withstand the mechanical stresses to a given extent, and for this reason, the elasticity of the synthesized glasses was theoretically evaluated by applying Makishima–Mackenzie model [59]. Young's, bulk and shear modulus (E, K and S) along with Poisson's ratio γ were calculated from the relations given below:

(17) $$E=2\;G_{t}\;V_{t}$$

$$K=1.2\;V_{t}\;E\;\left(18\right)$$

(19) $$S=\frac{3\;E\;K}{9K-E}$$

(20) $$\gamma =\frac{E}{2S-1}$$

Here, G_t_ denotes dissociation energy per unit volume corresponding to constituent oxides and *V*_*t*_ is their packing density. The values determined for different compounds are listed in the Table 3.

(21) $$\mathrm{Gt}=\sum_{i}G_{i}x_{i}$$

(22) $$\mathrm{Vt}=\frac{\rho }{M}\sum_{i}V_{i}x_{i}$$

**TABLE 3:** Dissociation energy and volume unit values calculated for the constituent compounds present in the synthesized co‐doped glasses.

Compounds	Dissociation energy G_i_ (×10^9^) Jmol^−1^ m^−3^	Volume unit *V*_*i*_ (×10^−6^) m^3^
B_2_O_3_	65.0332	19.895
SiO_2_	41.0804	13.3032
Eu_2_O_3_	23.3821	13.9841
Gd_2_O_3_	21.6644	24.173
TeO_2_	29.9058	24.052
Bi_2_O_3_	14.1103	12.8358
BaO	22.2209	7.1743
ZnO	21.4811	25.4012

Dissociation energy/unit volume:

(23) $$\mathrm{Gi}=\frac{\rho_{i}}{M_{i}}\left[X∆H_{f_{\left(M\;\mathrm{gas}\right)}}+Y∆H_{f_{\left(O\;\mathrm{gas}\right)}}-∆H_{f_{\left(M_{x}O_{y}\right)}}-\left(X+Y\right)\mathrm{RT}\right]$$

where $\rho_{i}$ and *M*_*i*_ denote density and molecular weight of the *i*th oxide, $∆H_{f}$ indicates molar heat formation and *R* and *T* are gas constant and room temperature respectively. Hess's cycle was utilized to calculate *G*_*i*_ values of B_2_O_3_, SiO_2_, TeO_2_, Eu_2_O_3_, Gd_2_O_3_, BaO, ZnO and Bi_2_O_3_ [60].

Density factor of each oxide:

(24) $$\mathrm{Vi}=\frac{4\pi N_{A}}{3}\left(x{r_{M}}^{3}+y{r_{O}}^{3}\right)$$

Here, $r_{M}$ and $r_{O}$ are the cationic and anionic radii, respectively. The estimated values of these elastic constants for EuGd‐0, EuGd‐1, EuGd‐2, EuGd‐3 and EuGd‐4 were tabulated in Table 4. There is enhancement of elastic moduli with the increase in Gd_2_O_3_ content directing towards the increasing mechanical strength with Gd^3+^ doping [25].

**TABLE 4:** Dissociation constant, packing density and elastic moduli of EuGd glass system.

Sample	G_t_ (×10^9^) Jmol^−1^ m^−3^	V_t_ (m^3^)	E (GPa)	K (GPa)	S (GPa)	ϑ
EuGd‐0	30.6115	0.5137	31.4503	19.3872	12.7885	0.2296
EuGd‐1	30.6761	0.5161	31.6639	19.6101	12.8622	0.2309
EuGd‐2	30.7413	0.5175	31.8172	19.7585	12.9169	0.2316
EuGd‐3	30.8065	0.5193	31.9956	19.9384	12.9794	0.2325
EuGd‐4	30.8718	0.5216	32.2055	20.158	13.0521	0.2337

## 4 Conclusion

The current work elaborates the influence of Eu^3+^–Gd^3+^ co‐doping on radiation shielding ability of glasses. Glass samples were prepared with different mol% of Gd_2_O_3_ using conventional melt quench process. 2 mol% Gd^3+^ co‐doping to the europium boro‐tellurite glass (EuGd‐2) provided the highest density value of 5.7405 gcm^−3^. The amorphous character of the co‐doped glasses was verified from XRD pattern. Radiation shielding parameters obtained from PSD software suggested that using Gd^3+^ and Eu^3+^ as co‐dopants substantially improved the gamma blocking ability of the glasses due to the enhanced density values. EuGd‐2 was the best possible composition in terms of LAC and HVL quantities. The HVL of EuGd‐0, EuGd‐1, EuGd‐2, EuGd‐3 and EuGd‐4 at 0.662 MeV were 1.48, 1.46, 1.38, 1.45 and 1.39 cm, respectively, with EuGd‐2 displaying the least HVL. The Z_eff_ and Z_eq_ values showed continuous enhancement with the increase in Gd_2_O_3_ content because of their dependence on molecular weight. ϒ‐ray photon build‐up study revealed that in the Compton scattering region, EuGd‐2 displayed minimum photon build‐up for penetration depths ranging from 0.5 to 40 mfp, contributing to better shielding property. EuGd‐2 glass also exhibited highest FNR probability (0.10935 cm^−1^) among the investigated glasses. When compared with the bismuth borosilicate, bismuth boro‐tellurite, singly doped Eu^3+^ and Gd^3+^ boro‐tellurite glasses, the EuGd‐2 glass displayed good competence in gamma radiation shielding with HVL following the order EuGd‐2 < BiTeGd‐4 < BiTeEu‐4 < BiTe‐40 < ZBiB‐12. Additionally, the co‐doping provided better quality of FNR in comparison to singly doped glasses. The absorption peaks corresponding to Eu^3+^ transitions were present in the UV–visible–NIR spectra. The mechanical investigation on the EuGd system confirmed that the Gd^3+^ doping provided better elasticity to the Eu^3+^‐doped glasses.

## Author Contributions

**Ashwitha Nancy D'Souza:** conceptualization, methodology, data curation, formal analysis, writing – original draft, investigation, validation, visualization. **Sumanth G. Madivala:** validation, data curation, writing – original draft. **A. Vidya Saraswathi:** resources. **M. I. Sayyed:** software, validation. **M. Rashad:** software, validation. **Sudha D. Kamath:** conceptualization, investigation, validation, formal analysis, project administration, supervision, resources, funding acquisition.

## Conflicts of Interest

The authors declare no conflicts of interest.

## Data Availability

The authors confirm that the data supporting the findings of this study are available within the article.
